# Effects of recording sequence on flicker electroretinographics recorded with natural pupils corrected for pupil area

**DOI:** 10.1111/aos.14618

**Published:** 2020-09-10

**Authors:** Asako Sugawara, Kumiko Kato, Ryunosuke Nagashima, Kengo Ikesugi, Masahiko Sugimoto, Hisashi Matsubara, Daphne McCulloch, Mineo Kondo

**Affiliations:** ^1^ Department of Ophthalmology Mie University Graduate School of Medicine Tsu Japan; ^2^ School of Optometry and Vision Science University of Waterloo Waterloo ON Canada

**Keywords:** flicker ERG, fundamental component, implicit times, pupil diameter, RET*eval*

## Abstract

**Background:**

A new handheld electroretinographic (ERG) recording system can record ERGs without mydriasis. However, this RET*eval* system cannot record ERGs from both eyes simultaneously. Thus, the purpose of this study was to determine whether the sequence of the ERG recordings will alter the results.

**Methods:**

We studied 30 eyes of 30 healthy subjects. The flicker ERGs were recorded with the RET*eval* system without mydriasis and were elicited by 8, 16 and 32 photopic Td‐s. The flicker ERGs were recorded at two sessions. Session 1, the ERGs were recorded from the right eye and then the left eye, and Session 2, ERGs were recorded from the left eye then the right eye. We compared the implicit times, amplitudes and pupil diameters of the right eye between these two sessions.

**Results:**

The implicit time of the flicker ERGs was significantly shorter (p < 0.001), and the pupil diameters were significantly smaller (p = 0.013) at Session 2 than Session 1 but only for the lower stimulus intensity of eight Td‐s. There was a significant correlation of the differences in the implicit times and the differences in the pupil diameter between the two sessions (*r* = 0.406, p = 0.026).

**Conclusions:**

The results indicate that the implicit times of the fundamental components of RET*eval* flicker ERGs can be affected by the sequence of recordings for lower stimulus intensities. This was most likely due to the differences of the pupil diameter during the recordings. We recommend that stronger stimuli be used to record the RET*eval* flicker ERGs to minimize the effects of the sequence of recordings.

## Introduction

Full‐field flicker electroretinographic (ERG) recordings can provide a rapid objective measure of retinal function. However, conventional ERG recording can be time‐consuming as pupillary mydriasis is used to standardize the amount of light falling on the retina. To streamline the testing process, a system called RET*eval^®^* (LKC Technologies, Gaithersburg, MD, USA) has been developed (Kato et al. 2015, 2017; Miura et al. 2017; Asakawa et al. 2017; Hobby et al, 2018; Liu et al. 2018). This system is comprised of a 6 cm, handheld Ganzfeld dome and a single skin‐electrode array called a sensor strip. With this system, full‐field flicker ERGs can be recorded under natural pupil conditions because the device delivers stimulus flashes with constant retinal illuminance (Td‐s) by adjusting the luminance (cd‐s/m^2^) to compensate for changes in the pupil area (mm^2^) in real‐time (Kato et al. 2015). The RET*eval* system can be used to evaluate the retinal function in eyes with diabetic retinopathy (Maa et al., 2016; Fukuo et al. 2016; Al‐Otaibi et al. 2017; Değirmenci et al. 2018; Zeng et al. 2019, 2020; Motz et al. 2020), central retinal vein occlusion (Yasuda et al. 2015; Miyata et al. 2018), inherited retinal dystrophy (Grace et al. 2017) and glaucoma (Tang et al. 2018a,b). This system can be especially useful when the physician wants to record the full‐field ERGs from children (Grace et al. 2017; Osigian et al., 2019; Tekavčič Pompe & Šuštar 2019) or just after intraocular surgery (Terauchi et al. 2019; Shibuya et al. 2019).

One disadvantage of a system with a handheld ganzfeld and real‐time pupil monitoring is that the ERGs cannot be recorded from both eyes at the same time because the small Ganzfeld dome does not cover both eyes at once; a larger dome design would create the technically complex issue of binocular pupil monitoring in real‐time. Hence, the examiners must always record the ERGs from one eye and then from the other eye. During our use of the RET*eval* system in the clinic, we questioned whether the ERGs recorded first are exactly the same as that recorded second. We raised this question because we have reported that the flicker ERGs recorded with RET*eval* system can be significantly affected by the pupil size (Kato et al. 2015). The pupil size during flicker ERG recordings may be different when the ERGs were recorded after the first recording.

Thus, the purpose of this study was to determine whether the flicker ERGs recorded first differs from that recorded second. To accomplish this, we compared the amplitudes and implicit times of RET*eval* flicker ERGs recorded first to that recorded second in normal subjects.

## Methods

### Study design

This was a prospective, single‐centre study conducted at the Mie University Hospital. The Medical Ethics Committee of Mie University Hospital (no. 2595) approved the procedures used, and the procedures conformed to the tenets of the Declaration of Helsinki of the World Medical Association.

### Subjects

We studied 30 healthy volunteers (14 men and 16 women; median age, 28 years; range, 20–56 years). All subjects had no known ocular or systemic diseases. All participants signed a written informed consent form after they were provided with information on the procedures and possible complications.

### Flicker ERG recordings by RET*eval* system

Full‐field flicker ERGs were recorded with the RET*eval* system. The RET*eval* system and analyses of the ERG waveforms have been described in detail (Kato et al. 2015). In brief, full‐field stimuli were presented with a 60‐mm diameter dome, and the visible ‘white’ stimuli were created by a combination of three‐coloured light‐emitting diodes. The device uses a built‐in infrared camera that measures the pupil size in real‐time and adjusts the flash luminance continuously to deliver a constant retinal illuminance throughout the measurement to the following equation:

$$\text{Photopic}\;\text{flash}\;\text{retinal}\;\text{illuminance}\;\;\left(\text{Td}‐s\right)\;\;=\;\text{photopic}\;\text{flash}\;\text{luminance}\;\;\left(\text{cd}‐s/m^{2}\right)\;\times \;\text{pupillary}\;\text{area}\;\left({\text{mm}}^{2}\right).$$

For this study, the flash retinal illuminance was set at 8, 16 or 32 photopic Td‐s. The flicker stimuli were presented at a rate of 28.306 Hz, and the pulse duration was less than 1 msecond. The flicker ERG recording time ranged from approximately 3–15 seconds depending on the time required to reach a phase stability criterion (RET*eval* ^®^ proprietary internal algorithm). Before the actual flicker ERG recordings, a pre‐recording with the flicker stimulus was presented to the eye to be recorded for about 3 seconds. The amplitudes and implicit times of the fundamental component were automatically measured and displayed by RET*eval* system using a special algorithm using discrete Fourier transformation (DFT) and cross‐correlation analysis (Kato et al. 2015). In this system, two flicker ERG waveforms, the fundamental component and the reconstructed flicker ERG waveform using the first eight harmonics are presented. We analysed the amplitudes and implicit times of the fundamental component of the flicker ERGs. We also calculated the mean pupil diameter (mm) during the flicker ERG recordings based on the data of the RET*eval* system.

### Experimental protocol

**Fig. 1:**
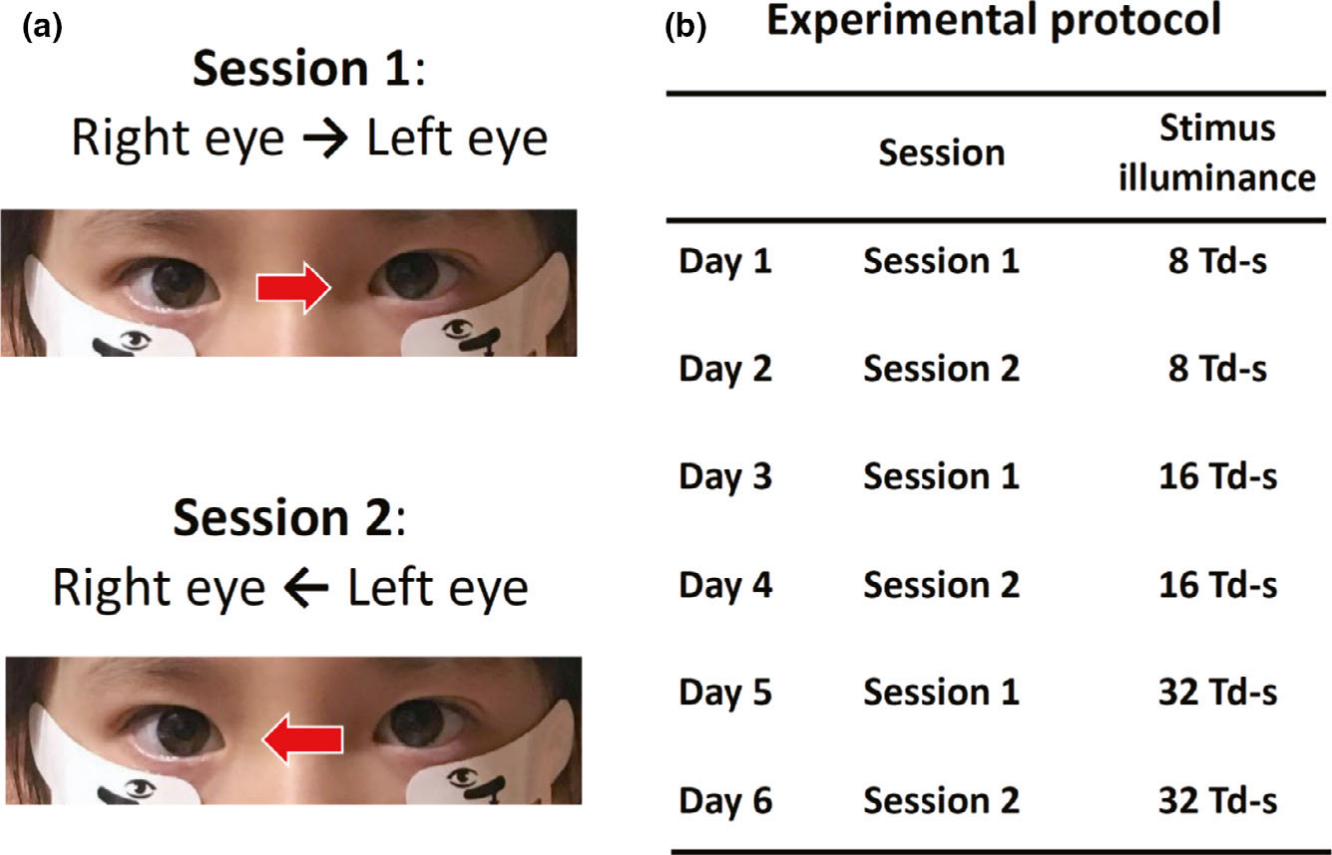
Two sessions used in this study and entire experimental protocol. (A) In Session 1, the flicker electroretinographics (ERGs) were recorded from the right eye then from the left eye. In Session 2, the flicker ERGs were recorded from the left eye first then the right eye second. (B) The entire experimental protocol. The RET*eval* flicker ERGs were recorded on six different days for each subject.

The flicker ERGs were recorded at two different sessions. In Session 1, ERGs were recorded from the right eye and then the left eye within 10 seconds. In Session 2, ERGs were recorded from the left eye then within 10 seconds from the right eye (Fig. 1A,B).

The flicker ERGs were recorded on six different days for each subject. The flicker ERGs at eight photopic Td‐s for Session 1 were recorded on day 1, and the flicker ERGs at eight photopic Td‐s for Session 2 were recorded on day 2. The flicker ERG at 16 photopic Td‐s for Session 1 was recorded on day 3, and the flicker ERG at 16 photopic Td‐s for Session 2 was recorded on day 4. The flicker ERG at 32 photopic Td‐s for Session 1 was recorded on day 5, and the flicker ERG at 32 photopic Td‐s for Session 2 was recorded on day 6.

The data of only the right eye were used for the analyses. The implicit times, amplitudes and pupil diameters during the ERG recordings of the right eye were compared between Session 1 and Session 2 for each flicker strength.

### Statistical analyses

Paired *t‐tests* were used to determine whether there were significant differences in the implicit times, amplitudes and pupil diameters between Session 1 and Session 2. The Pearson product–moment correlation coefficient was used to determine whether there was a significant correlation between the differences of the pupil diameter between the two sessions and difference of implicit time of flicker ERG between the two sessions. The results were considered statistically significant when p was <0.05.

## Results

### Demographic data of 30 healthy subjects

We studied 30 eyes of 30 healthy subjects. The subjects were 14 men and 16 women, and the mean age was 29.8 ± 7.1 years. The decimal best‐corrected visual acuity was ≥1.0 for all subjects. The mean axial length was 25.0 ± 0.9 mm (range 23.4–26.8 mm).

### Representative flicker ERG waveforms

**Fig. 2:**
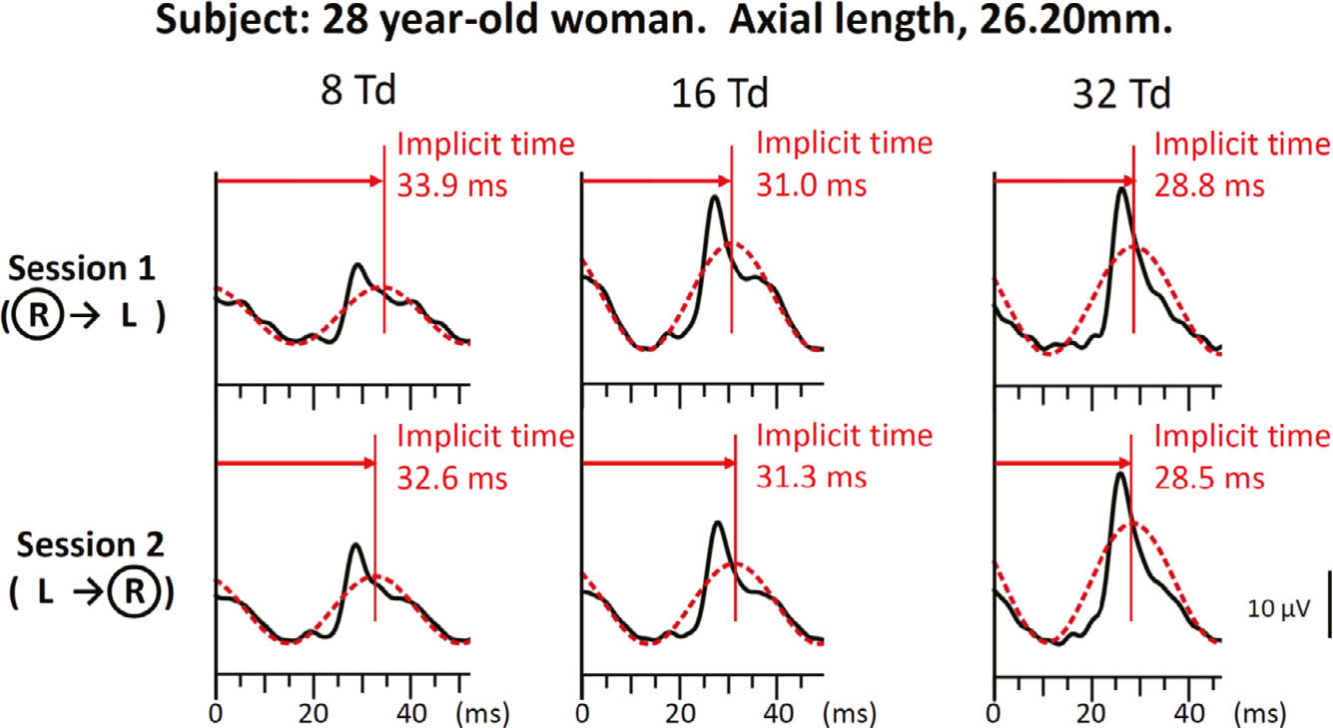
Representative RET*eval* flicker electroretinographic (ERG) waveforms recorded from a representative 28‐year‐old woman during Session 1 and Session 2. Only the ERG waveforms of the right eye are shown. The fundamental component (dotted red line) is superimposed on the reconstructed flicker ERG waveform using the first eight harmonics (solid black line). Red vertical lines are drawn at the implicit times of the fundamental component. The implicit times of the fundamental component were shorter for Session 2 than for Session1 when the weaker stimulus intensity of 8 photopic Td‐s was used (left panel). There was no obvious difference in the implicit times between the two sessions for the stimulus intensity of 16 and 32 photopic Td‐s.

Representative flicker ERGs recorded from a normal subject (28‐year‐old woman) are shown in Fig. 2. Only the ERG waveforms recorded from the right eye are shown. The implicit time of the fundamental component became shorter when the flicker ERG was recorded after the initial ERG of left eye for eight photopic Td‐s (left panel of Fig. 2). There were no obvious differences in the amplitudes between the two sessions for eight photopic Td‐s. There were also no apparent differences in the implicit times and amplitudes for 16 and 32 photopic Td‐s (middle and right panels of Fig. 2).

### Implicit times, amplitude of flicker ERGs and pupil diameter during ERG recordings

**Fig. 3:**
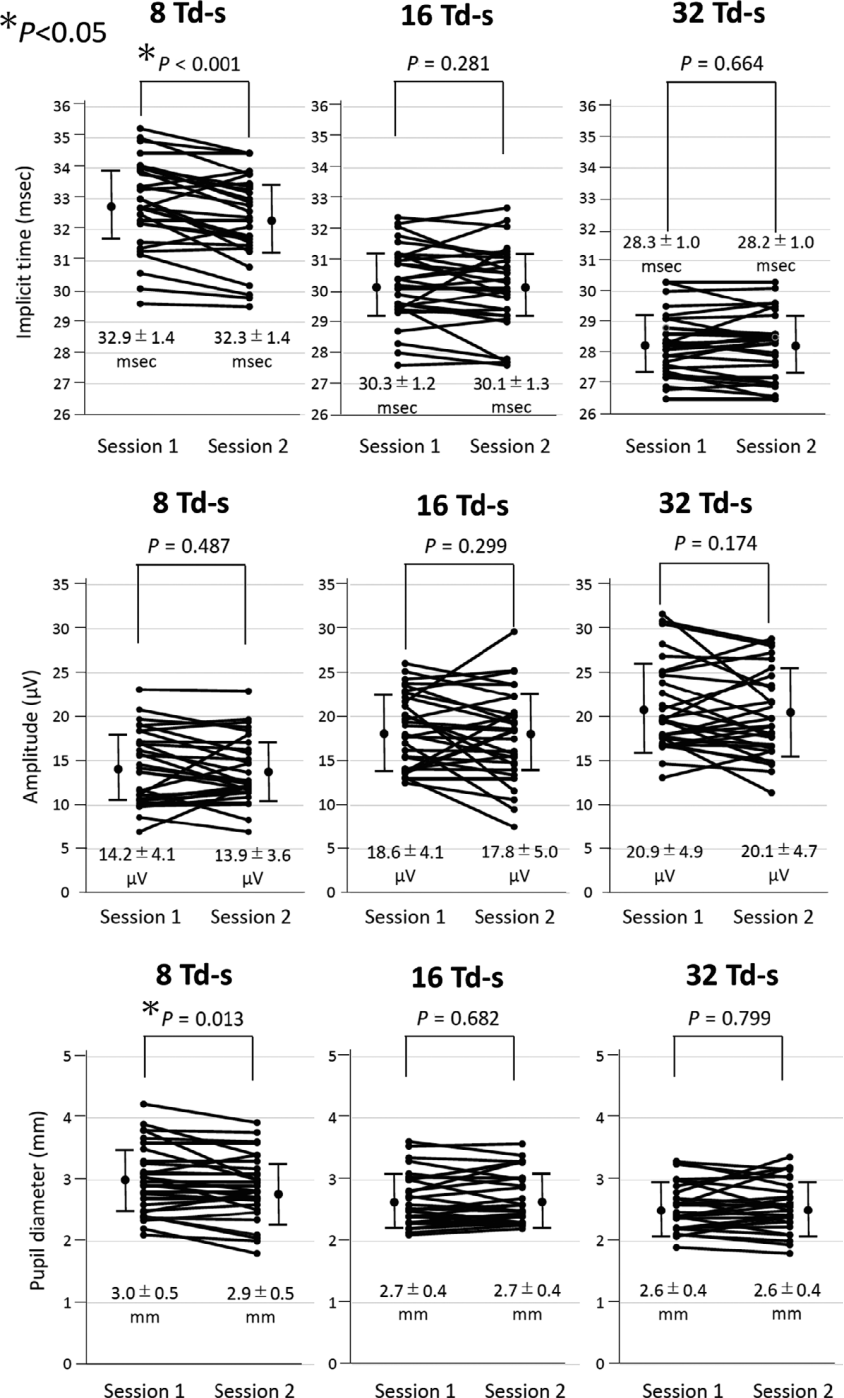
Comparisons of the implicit times, amplitudes and pupil diameters during flicker electroretinographics (ERGs) recording between Session 1 and Session 2. The implicit times of the fundamental component were significantly shorter for Session 2 than for session 1 when the flicker ERGs were recorded with the stimulus intensity of 8 photopic Td‐s (p < 0.001, upper left panel). Pupil diameter during flicker ERG recording was also significantly smaller for session 2 than session 1 for stimulus intensity of 8 photopic Td‐s (p = 0.013, lower left panel).

The mean (±SD) of the implicit times, amplitudes and average pupil diameters during the flicker ERG recordings in Session 1 and Session 2 for the three different stimulus intensities are plotted in Fig. 3. Only the values of the right eye are shown. The implicit time of the fundamental component of the flicker ERGs was significantly shorter (p < 0.001), and the average pupil diameter during the ERG recordings was significantly smaller when the flicker ERGs were recorded second than those recorded first for the weakest stimulus intensity of eight photopic Td‐s (p = 0.013). There was no significant difference in the amplitudes of the fundamental components of the flicker ERGs recorded with eight photopic Td‐s between the two sessions. There was also no significant difference in the implicit times, amplitudes and pupil diameters during the ERG recordings between the two sessions for 16 and 32 photopic Td‐s. We also analysed the data of the left eye and found that the results of the left eye were almost the same as those of the right eye (data not shown).

### Relationship between implicit times of flicker ERG and pupil diameter

**Fig. 4:**
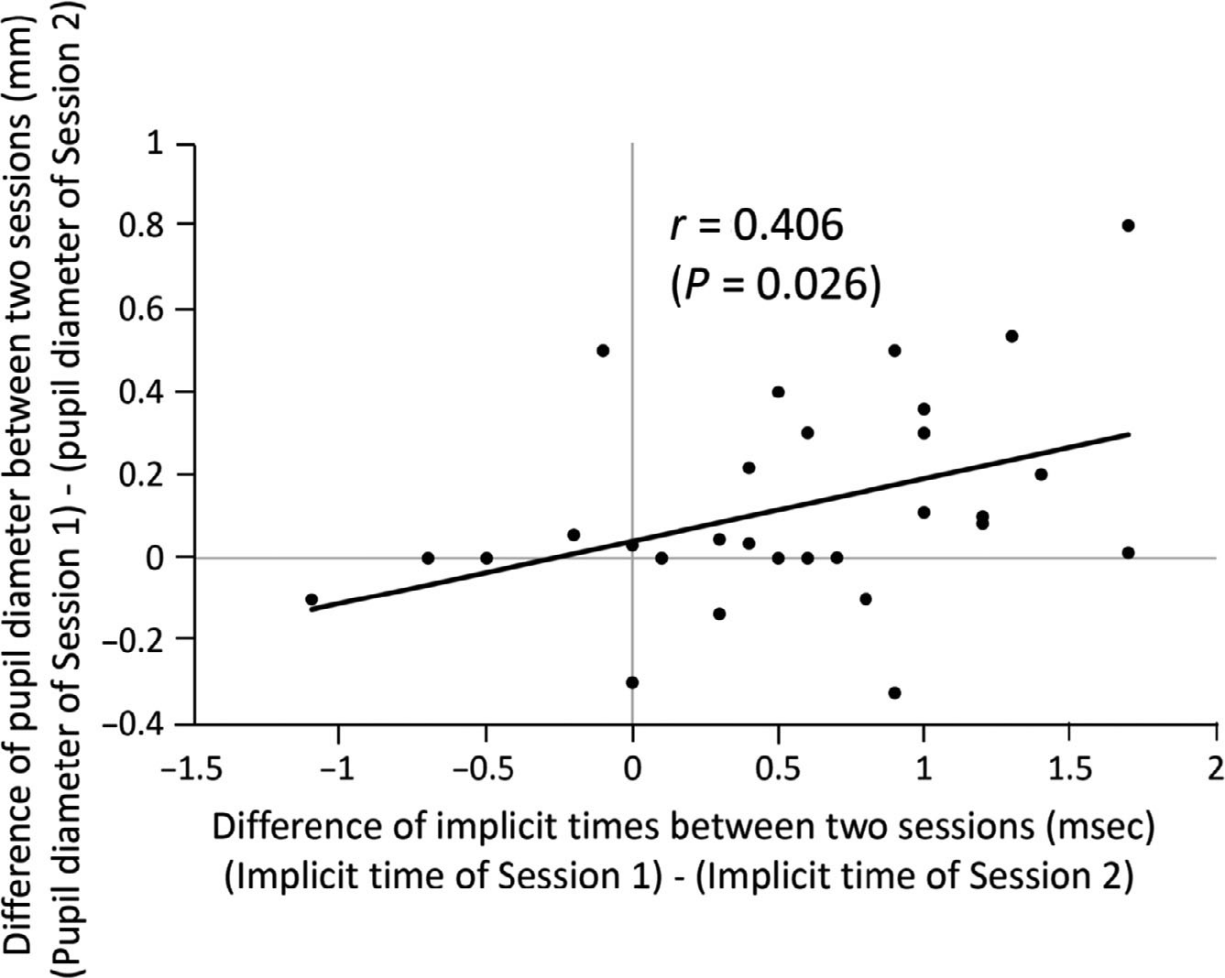
Plots of the differences of the pupil diameter during the flicker electroretinographic (ERG) recordings between the two sessions (pupil diameter of session 1 minus pupil diameter of session 2) against the difference of implicit times between the two sessions (implicit time of session 1 minus implicit time of session 2). There was a weak, but significant, correlation between these two factors (*r* = 0.406, p = 0.026).

To determine whether the difference in the implicit times between the two sessions for eight Td‐s was related to the pupil diameter during the recordings, we plotted the difference of implicit times between two sessions, that is implicit time of Session 1 minus implicit time of Session 2, against the difference of pupil diameter between the two sessions, that is pupil diameter of Session 1 minus pupil diameter of Session 2, for stimulus intensity of eight photopic Td‐s (Fig. 4). We found that there was a weak, but significant and positive, correlation between the difference of implicit times between the two sessions and the difference of pupil diameter between the two sessions (*r* = 0.406, p = 0.026).

We also investigated the correlation between the difference of implicit times between two sessions and the difference of pupil diameter between two sessions for 16 and 32 Td‐s, but there were no significant correlations.

### Changes in pupil diameter during ERG recordings

**Fig. 5:**
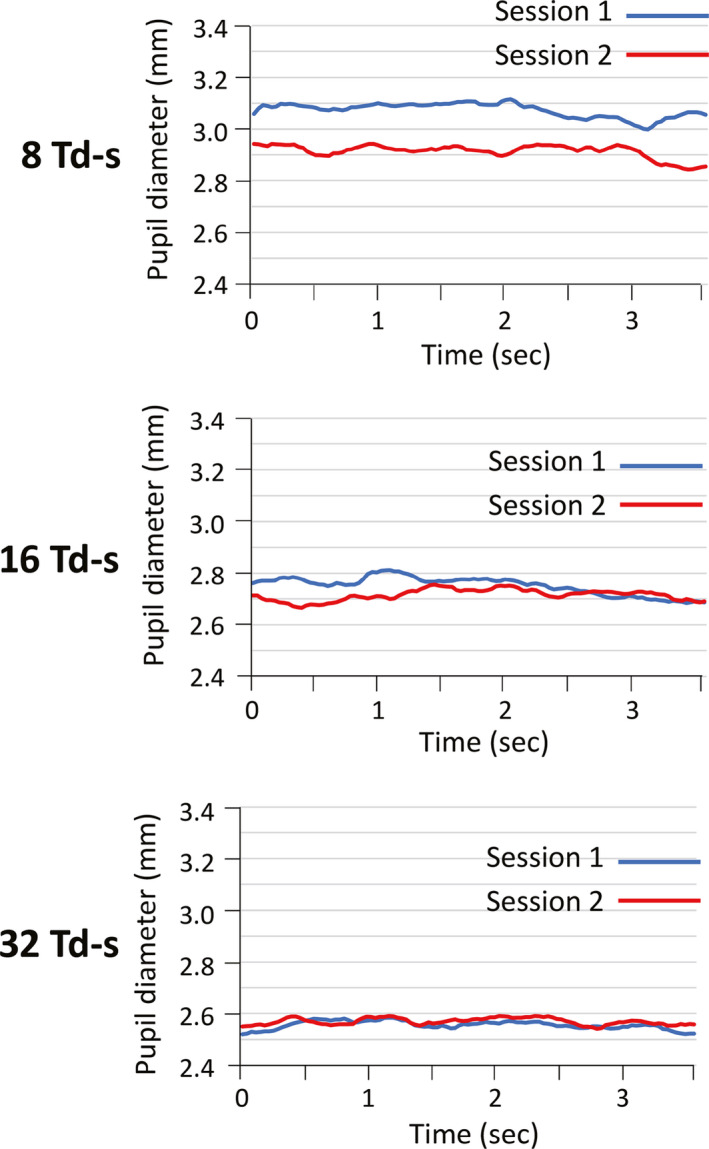
The actual changes in pupil diameter during flicker electroretinographic (ERG) recordings. The average pupil diameter during flicker ERG recording for all 30 subjects is plotted against time. Because we noted that the shortest recording time of the flicker ERGs among the 30 normal subjects was 3.64 seconds, the average pupil diameters for 30 normal subjects until 3.64 seconds are shown. We found that the pupil diameter tended to be larger for Session 1 than for Session 2 during all ERG recording time for the lowest stimulus intensity of 8 Td‐s (top panel). This difference in pupil diameter between two sessions is smaller for the intermediate and higher stimulus intensities of 16 and 32 Td‐s, respectively.

Finally, we investigated the actual changes in the pupil diameter during the flicker ERG recordings for all sessions. We have plotted the average pupil diameter during the flicker ERG recordings for all 30 subjects. As described in the Methods, the total flicker ERG recording times were different among the 30 normal subject due to blink and baseline artefacts. Because we noted that the shortest recording time of flicker ERG among 30 normal subjects was 3.64 seconds, the average pupil diameters for 30 normal subjects until 3.64 seconds are plotted. We found that the pupil diameter tended to be larger for Session 1 than for Session 2 during all ERG recording times for lowest stimulus intensity of eight Td‐s (top panel of Fig. 5). However, this difference in the pupil diameter between the two sessions became smaller for the intermediate stimulus intensity of 16 Td (middle panel of Fig. 5). For highest stimulus intensity of 32 Td‐s, there seemed to be no apparent difference in the pupil diameter between the two sessions (lowest panel of Fig. 5).

## Discussion

Our results demonstrated that the implicit time of the fundamental component of the RET*eval* flicker ERGs recorded second was significantly shorter than that of the first recording but only for the weakest stimulus of eight photopic Td‐s (p < 0.001, Figs 2 and 3). For stronger retinal illuminances of 16 and 32 Td‐s, there was no significant difference in the implicit times between two the sessions (p > 0.2, Fig. 3). These results suggest that the implicit time of the fundamental component of the RET*eval* flicker ERGs can be affected by the sequence of recordings, but only when the eight photopic Td‐s stimuli were used.

Why were the implicit times of the fundamental component of flicker ERGs shorter when the flicker ERGs were recorded second than when recorded first but only for the eight Td‐s stimulus? There is no simple explanation for the effect of test order for only one of three flicker stimuli. However, we suggest that the shorter implicit times are associated with smaller pupil diameters during the ERG recordings. As shown in Fig. 3, the pupil diameters were significantly smaller in Session 2 than in Session 1 for eight Td‐s stimuli (lower left panel), and there was a significant correlation between the differences of the implicit times between the two sessions and the difference of the pupil diameter between the two sessions (Fig. 4).

The adjustment of the stimulus flash luminance according to pupil area to produce equal retinal illuminance, that is equal Troland values of luminance × pupil area, assumes that the entire pupil area to be equally effective. However, the cone outer segments are directionally sensitive such that light entering the central pupil region is more effective than light entering obliquely through peripheral regions of the pupil, a property known as the Stiles–Crawford effect (McIntyre & Pask. 2013). Thus, equal retinal illumination calculated for a small pupil will be more effective than that calculated for a larger pupil. We have demonstrated that the implicit times of flicker ERGs are longer for those with larger pupil areas, although the Troland values were equalized using the RET*eval* system (Kato et al. 2015). Therefore, we believe that the larger pupil diameter in Session 1 is associated with the prolonged implicit time in Session 1 for eight Td‐s stimuli.

The implicit time of flicker ERGs has a complex relationship with the strength of the flicker stimulation. When a very wide range of stimuli are used, implicit times are shortest for moderate luminance levels (around 30 Td‐s) and show an increase for both higher and lower luminance stimuli, resulting in an inverted U‐shaped function (Peachey et al. 1992). Thus, for stimuli weaker than 30 Td‐s, including our eight Td‐s stimulus, a stronger effective flicker stimulus through a smaller pupil is expected to generate flicker ERGs with shorter implicit times in keeping with our previous studies (Kato et al. 2015, 2017).

The question then arises as to why the difference in pupil size between two sessions was seen only for the weaker stimulus of eight Td‐s, but not for the stronger stimuli of 16 and 32 Td‐s (Figs 3 and 4, lower panel). We found that the pupil diameter tended to be larger for Session 1 than for Session 2 during all ERG recordings. However, for the intermediate and stronger stimuli of 16 and 32 Td‐s the small difference in pupil diameter between two sessions did not reach significance (Fig. 5). For the weaker stimulus, it is likely that the pupils were smaller during the second recording due to a phenomenon known as the post‐illumination pupil response (PIPR), sustained pupil constriction for up to several minutes following indirect pupil illumination. The size and duration of the PIPR depend on the strength and duration of the illumination (Adhikari et al. 2015). Although the PIPR has not been quantified for flickering stimuli, it is reasonable that the PIPR caused smaller pupil diameters during the second recordings following the continuous flicker stimuli during the ERG recordings to the first eye.

We suggest that for the stronger stimuli of 16 and 32 Td‐s, the PIPR may have saturated during 3 seconds pre‐recording to the stronger flicker stimuli leading to nearly equal pupil diameters in the first and second recordings. In contrast, for the weaker stimulus of 8‐Td, the direct pupillary response in the second eye might have added to the indirect PIPR from testing of the first eye resulting is a smaller pupil.

We used three different retinal illuminances of 8, 16 and 32 photopic Td‐s, because these three were the default stimulus settings for flicker ERG recordings under natural pupillary conditions. However, based on the present results, we believe that the use of weaker retinal illuminances of eight Td‐s may not be appropriate for RET*eval* flicker ERG recordings in the clinical situation because the implicit time of fundamental component can be affected by the sequence of recordings. In fact, the eight photopic Td‐s stimulus was much weaker than the ISCEV standard for flicker stimulation which is approximately 150 Td‐s (McCulloch et al. 2015). Therefore, we recommend the use of the strongest retinal illuminance of 32 Td‐s for the RET*eval* flicker ERG recordings because both the implicit time of fundamental component and the pupil diameter during flicker ERGs were less affected by the sequence of recordings (Figs 3 and 5).

There are limitations in this study. The first limitation is that we analysed mainly the fundamental component of the flicker ERG, although the measurements of the peak implicit times or peak‐to‐peak amplitudes to the flicker ERG including their harmonics are more generally used in clinical situations. This was simply because the implicit times and amplitudes of the fundamental component are automatically displayed in the RET*eval* system. In fact, we have examined the difference in the peak implicit times and peak‐to‐peak amplitudes for the waveform with the harmonics between Session 1 and Session 2 and found similar results with longer implicit times for the larger pupils found for the eight Td‐s flicker (Fig. S1).

A second limitation is that our protocol did not consider the dynamics of PIPR. Pupil re‐dilation to baseline depends not only on the strength and duration of the stimulus but also on the wavelength of the stimulus with the maximal effect for melanopsin‐sensitive ganglion cells which regulate pupillary responses. It is possible that avoiding the melanopsin sensitivity using of longer wavelength flicker stimuli or simply pausing the test between eyes may negate the second eye effects.

The third limitation is that the number of subjects might not be enough. The amplitude of the ERG seems to be reduced in the session 2 under all the stimulus conditions, although it was not statistically significant. There might be a possibility that the sequence of recording might affect not only the implicit times but also the amplitudes especially with the higher stimulus intensities. We are planning to increase the number of subjects and re‐analyse the difference in amplitude between two sessions.

Finally, we studied only the flicker ERGs and did not study how the sequence of recordings affected the other ERG components such as the rod response, maximal rod and cone response, and single‐flash cone response in the RET*eval* system under natural pupils. We are now planning to study this in the next project.

In conclusion, we found that the implicit times of fundamental component of the RET*eval* flicker ERGs became shorter when recorded second than recorded first for weaker stimulus of eight Td‐s. Our results suggested that this phenomenon was caused by the difference in the pupil diameter during the ERG recordings. We recommend the use of stronger stimuli for RET*eval* flicker ERG recordings under natural pupil conditions to minimize the effect of sequence of ERG recordings.

## Supporting information

**Figure S1.** Comparisons of the peak implicit times and peak‐to‐peak amplitudes during flicker ERGs between Session 1 and Session 2. The peak implicit times was significantly shorter for Session 2 than for session1 when the flicker ERGs were recorded with the stimulus intensity of 8 photopic Td‐s (p = 0.008, upper left panel).
